# The Dental Management of Patients at Risk of Medication-Related Osteonecrosis of the Jaw: New Paradigm of Primary Prevention

**DOI:** 10.1155/2018/2684924

**Published:** 2018-09-16

**Authors:** Olga Di Fede, Vera Panzarella, Rodolfo Mauceri, Vittorio Fusco, Alberto Bedogni, Lorenzo Lo Muzio, SIPMO ONJ Board, Giuseppina Campisi

**Affiliations:** ^1^Department of Surgical, Oncological and Oral Sciences, University of Palermo, Palermo, Italy; ^2^Oncology Unit, SS Antonio e Biagio e Cesare Arrigo Hospital, Alessandria, Italy; ^3^Unit of Maxillofacial Surgery, Department of Neurosciences (DNS), University of Padua, Padua, Italy; ^4^Department of Clinical and Experimental Medicine, University of Foggia, Foggia, Italy; ^5^Italian Society of Oral Pathology and Medicine (SIPMO), Foggia, Italy

## Abstract

Medication-related osteonecrosis of the jaw (MRONJ) is a serious adverse reaction of antiresorptive and antiangiogenic agents; it is a potentially painful and debilitating condition that can considerably affect the quality of life of patients. Furthermore, even if its epidemiology and pathogenesis have still not been fully clarified, several risk factors related to MRONJ have been recognized in prevention protocols. Three main risk factors are as follows: (i) the type of ONJ-related medications: antiresorptive (e.g., Bisphosphonates, Denosumab) and antiangiogenic drugs (e.g., Bevacizumab, Sunitinib); (ii) the category of patient at MRONJ risk: cancer versus non-cancer patient; (iii) the typologies and timing of dental treatments (e.g., before, during, or after the drug administration). The aim of this paper is to describe the new paradigm by the Italian Society of Oral Pathology and Medicine (SIPMO) on preventive dental management in patients at risk of MRONJ, prior to and during/after the administration of the aforementioned ONJ-related drugs. In reducing the risk of MRONJ, dentists and oral hygienists are key figures in applying a correct protocol of primary prevention for pre-treatment and in-treatment patients. However, the necessity of a multidisciplinary standardized approach, with a sustained dialogue among specialists involved, should be always adopted in order to improve the efficacy of preventive strategies and to ameliorate the patient's quality of life.

## 1. Introduction

Medication-related osteonecrosis of the jaw (MRONJ) is a relatively rare but potentially serious and debilitating complication. It consists of progressive bone destruction in the maxillofacial area of patients exposed to the treatment with drugs associated with the risk of ONJ, in the absence of a previous radiation treatment [1–4]. The diagnosis of MRONJ is based on the patient's pathological and pharmacological history and on the clinical and radiological features of progressive bone destruction (both exposed and not exposed) [1, 5].

MRONJ epidemiology and pathogenesis are still unclear; however, in recent years, notable progress has been made regarding the prevention of MRONJ by studying local risk factors (e.g., presence of infective, dental-periodontal, and/or peri-implant disease) in patients at risk of MRONJ and by planning dental procedures [1, 5–9].

Primary prevention, whose main aim is the elimination of oral and dental risk factors, is targeted at restoring and/or maintaining good oral health and reducing the risk of an onset of pathological conditions or any other negative event. This approach has the greatest impact when aimed at protecting constantly the patient's oral health, which is at risk of MRONJ by virtue of controlling local related risk factors [6, 9].

Subsequent to the initial reporting of MRONJ, fifteen years ago, attention has been focused on the association between dental extraction and adverse event in patients who were already being treated with ONJ-related drugs [10–12]. More recently, the presence of infection at dental-periodontal and peri-implant locations has been underlined as being one of the main local risk factors of developing MRONJ, often being the main reason of surgical procedures of dental extraction or implant removal [1, 5, 13]. The link between periodontal disease and the development of MRONJ has been widely demonstrated, and the spreading of bacteria via the periodontal pockets is one of the main mechanisms for transmitting infection throughout the alveolar bone. Indeed, it is not only the presence of* Porphyromonas gingivalis* in the periodontal pockets but also IgG products which probably promote the development of MRONJ. The concurrent action of* P. gingivalis* and IgG products would appear to increase bone remodeling and contemporaneously produce pro-inflammatory effects, thereby reducing the healing process of periodontal tissue and encouraging the development of MRONJ. Therefore, a severe periodontitis will be determinant for a poor prognosis for the teeth, for whom the only resolution is extraction; the latter has already been discussed as a further trigger for the developing of MRONJ [1, 7, 8, 14–19].

Many studies have demonstrated how, prior to commencing treatment with ONJ-related drugs, dental screening and treatments of oral diseases can significantly reduce the occurrence of this adverse event [9, 20–22]. Already in 2009, Dimopoulos et al. underlined the importance of dental management in patients eligible for treatment with ONJ-related drugs; this primary preventive measure subsequently produced a reduction by one-third of the incidence of MRONJ in the enrolled patients [20]. Similar results have also been obtained from other research groups, which highlighted the crucial role played by the physician and the dentist in the primary prevention [9, 21–23]. It is the responsibility of the physician to provide all relevant information regarding the risk of developing MRONJ for patients who are about to commence treatment with antiresorptive (AR) and/or antiangiogenic (AA) drugs. Moreover, it is also the physicians' duty to advise the patients about the relevance of an examination by an oral health specialist with the aim of assessing the necessity for preventive dental management. This should be performed prior to commencing, during and also after the treatment with ONJ-related drugs, in order to eliminate any infective outbreaks of MRONJ [14, 24, 25]. It is the responsibility of the dentist to accurately assess risk factors leading to the development of MRONJ and suggest a strategy for removing these factors. The dentist must also stress the importance of maintaining effective dental hygiene, including regular check-ups, for the patient. Both are necessary for maintaining oral health, reducing the outbreak of MRONJ, and/or detecting possible signs of the early symptoms of this disease.

## 2. Variables of Patients at Risk of MRONJ

When planning primary prevention measures, every specialist assigned to the care and maintenance of oral health must bear in mind three variables related to the assessment of the risk of MRONJ.

### 2.1. The Activity of ONJ-Related Drugs: Antiresorptive and Antiangiogenic Drugs

Drugs involved in the etiopathogenesis of MRONJ belong basically to two main categories: those with a mainly antiresorptive property and those with mainly antiangiogenic property. The AR drugs includes the following:

- Bisphosphonates (BPs), synthetic analogues of pyrophosphates, which firmly bind to the hydroxyapatite and reduce bone metabolism/remodeling. They are long half-life medications, which constitute a determining variable for their residual power in a “non-active” form in bone tissue after treatment has been interrupted. Indeed, the half-life of BPs in circulation is quite short, ranging from 30 minutes to 2 hours; however, once they have been incorporated into bone tissue, they can persist more than 10 years, depending on the skeletal turnover time [25].
- Denosumab, a monoclonal human IgG2 antibody that highly binds the* receptor activator of nuclear factor-kB ligand *(RANK-L), blocks the osteoclast maturation, function, and survival. It has a half-life of 25–32 days [26, 27].

Of the drugs related to a risk of MRONJ with a main AA activity, the most common are Vascular Endothelial Growth Factor (VEGF) inhibitors (e.g., Bevacizumab), tyrosine-kinase inhibitors (TKIs) (e.g., Sunitinib), and the mammalian target of Rapamycin (mTOR) inhibitors (e.g., Everolimus) [3]. AA drugs are generally indicated only for the treatment of oncological pathologies, by inhibiting the various mechanisms involved in tumour neoangiogenesis. Moreover, as is the case with Denosumab, these drugs do not tend to accumulate in the bone, and they have a well-known half-life, which varies on the basis of the molecule (from 30 hours for Everolimus to 20 days for Bevacizumab) [3].

### 2.2. The Categories of Patients at Risk: Cancer or Non-cancer

On the basis of epidemiological data regarding the onset of MRONJ, the risk is greater for cancer patients, who are probably contemporaneously exposed to a high number of MRONJ risk factors [1, 5, 13]. Indeed, there is a frequency of adverse event between 0.2% and 6.7% in cancer patients exposed to ONJ-related drugs, while the risk of developing MRONJ in patients affected by osteometabolic diseases, such as osteoporosis, is very low, with a prevalence between 0% and 0.4% [5]. However, due to the huge number of patients in the world affected by osteometabolic diseases, in terms of frequency, approximately 40% of patients affected by MRONJ are non-cancer patients [28].

### 2.3. Typologies and Timing of Dental Treatments 

The primary purpose of preventive dental approach is the detection and management of local risk factors related to MRONJ (Table 1). Due to the risk/benefit ratio of dental procedures and the risk of MRONJ, it is useful to distinguish three typologies of dental treatments: (i)* indicated*, which are necessary to prevent the risk of MRONJ; (ii)* possible*, which are considered irrelevant in regard to the risk of MRONJ; (iii)* contraindicated*, which are associated with a recognized risk of MRONJ.

This distinction will be better detailed in the following paragraphs also with respect to patients' categories (cancer versus non-cancer patients) and to ONJ-related drugs exposure time. About the timing of dental procedures, patients can be divided into two categories:

If the patient has never taken ONJ-related drugs, also called in the pre-treatment phase, the patient's oral health must be precisely assessed by clinical and radiographic examinations (mandatory for cancer patients), in order to evaluate the patient's dental-periodontal status and to plane the adequate dental therapies compatible with systemic diseases and the oncologist/physician's opinion.

If the patient has already been exposed to ONJ-related drugs, hereafter defined as the in-treatment phase, this patient will be included in an assessment program of oral health, the aim of which is to obtain and maintain as low as possible the level of local risk factors for MRONJ.

It will be necessary to continually remind the patient of the necessity both of maintaining effective oral hygiene at home, via counselling strategies, and of monitoring early clinical signs or symptoms of MRONJ. Dental procedures for the patient (both cancer and non-cancer) in pre-treatment and in-treatment phases will now be discussed in order to enhance our understanding of this topic. Moreover, invasive and non-invasive dental procedures will be also differentiated on the basis of above mentioned distinction (*indicated, possible, contraindicated*) (see Tables 2 and 3). Of great assistance in this regard is the use of leaflets, such as those which can be downloaded from https://www.unipa.it/dipartimenti/di.chir.on.s./.content/documenti/ONJ-Leaflet-_SIPMO-by-Di-Fede-Campisi20-02-17.pdf. Additionally, the app DoctOral® provides an open-access consultation of guided paths and recommendations regarding the dental management of patients at risk of MRONJ; it is free available both for android (https://play.google.com/store/apps/details?id=com.olgadifede.olgapp&hl=it) and iOS system (https://itunes.apple.com/us/app/doctoral/id1071070334?l=it&ls=1&mt=8) [29].

## 3. Cancer Patients in the Pre-treatment Phase

In the pre-treatment phase, cancer patients with good oral health must be informed and made aware of the risks inherent of MRONJ and the necessity of being enrolled onto a program with a 4-month follow-up, in order to monitor the status of the hard and soft tissues. Moreover, the patient should be encouraged to follow specific measures regarding secondary prevention (early recognition of the disease) and be informed about oral hygiene at home via counselling.

In cancer patients with tooth with poor or hopeless prognosis or other dental-periodontal infection, it would be desirable to defer the commencing of ONJ-related drugs after the tissue involved in any invasive dental treatment has healed. This includes at least the healing period of the soft tissue, which is usually approximately 45–60 days, prior to commencing AR/AA cancer treatment. For all other non-invasive dental procedures whose outcome is reliable, it is not necessary to defer cancer treatment. If cancer treatment cannot be delayed and invasive dental procedures are needed, it will be necessary to consider the patient as already being in treatment phase. Thereafter, the protocols of medical and surgical prophylaxis must be applied (see Section 5) [14, 25].

In greater detail, dentoalveolar surgeries are considered to be* indicated* invasive dental procedures; it would be convenient to reduce to a minimum any bone manipulation and encourage primary intention healing.

Other invasive procedures (e.g., implant surgery, pre-implant bone surgery, and mucogingival surgery) are* contraindicated*, since these are not aimed at the elimination of infection and they have often a rehabilitation/aesthetic aim; moreover, anyway these procedures will have an undefined long-term risk of developing MRONJ after the administration of ONJ-related drugs [14, 25, 26]. Dental treatments in cancer patients in the pre-treatment phase are described in Table 2.

## 4. Non-cancer Patients in the Pre-treatment Phase

Similarly, in this group of patients the primary objective is to maintain and/or reestablish as soon as possible an acceptable level of oral health, possibly before the administration of AR drugs or within its first six months [14]. If the patient presents with good oral health, it is beneficial to plan a six-month follow-up examination in order to maintain the primary prevention program.

In general, in a given non-cancer patient in the pre-treatment phase, surgical and non-surgical dental procedures are classified as* indicated* if regarding the treatment of infective conditions (e.g., dental-alveolar surgery, surgical and non-surgical endodontics, and surgical and non-surgical periodontics). All elective procedures (e.g., prosthetic rehabilitation with/without dental implant, or orthodontic treatment) are classified as* possible* with unknown or indefinable low risk of MRONJ. Dental treatments in non-cancer patients in the pre-treatment phase are described in Table 2.

## 5. Cancer Patients in Treatment Phase

From the first assumption of ONJ-related drugs for treating cancer, the patient is considered to be at a high risk of developing MRONJ [1, 5, 13, 14, 25, 27]. This is due to the contemporaneous presence of known, multiple risk factors.

Surgical procedures which are necessary for eliminating infective outbreaks of MRONJ are defined as* indicated* for cancer patients in-treatment in presence of dental diseases which cannot otherwise be resolved [1, 14].

The protocol regarding the dental extractions in cancer patients at risk of MRONJ promoted by the Italian Society of Oral and Maxillofacial Surgery (SICMF) and the Italian Society of Oral Pathology and Medicine (SIPMO) combines a medical prophylaxis with strictly surgical procedures. An example of a standardized protocol for dental extractions expects a medical prophylaxis that includes a 0.12% chlorhexidine (CHX) antiseptic mouthwash to be used at home 3 times a day, starting from 7 days prior to the planned dental procedure, associated with an antibiotic therapy (e.g., Ampicillin/Sulbactam im and Metronidazole per os) that must be administered from the day before the intervention and for at least 6 days following intervention. During the surgical procedures, it is advisable to use local anesthesia without adrenaline, to perform a full thickness flap, to gently remove the tooth, to do the alveoloplasty of the postextraction site (if necessary), and to apply a tension-free soft tissue closure, to promote the healing by first intention[26]. Moreover, the use of ultrasound surgical equipment is preferable for bone manipulation, even if, currently, conventional dental instruments do not seem to increase the risk of MRONJ, notwithstanding their more invasive nature [14, 30, 31].

The post-operative medical therapy will be accompanied by a topical one, CHX mouthwash (3 times a day for 15 days), and growth-promoting treatment, as gel containing hyaluronic acid (three times per day for 15 days) [14, 26]. Sutures can be removed between the seventh and tenth day after intervention. Thereafter, periodic clinical check-up should continue with an accurate time schedule (at 3, 6, and 12 months) during the first year of follow-up.

When several dental extractions are necessary, it would be desirable to proceed one tooth at a time, particularly when ONJ-related drugs have not been suspended. Recently, surgical proposals have been considered, which also deploy a low-level, Nd:YAG laser and/or autologous platelet concentrates (APCs) [32, 33]. The application of APCs [34] with enhanced stability (e.g., plasma rich in growth factor (PRGF) and leucocyte-platelet-rich fibrin (L-PRF)) is yielding promising results in reducing the incidence of MRONJ following a dental extraction, thereby reducing the operating time and the extent of necessary surgery mucocele [34, 35].

When inflammatory-infective processes may be treated with periodontal and/or endodontic surgical procedures, the clinician should apply the same recommendations regarding dental extractions, and this also concerns medical prophylaxis and minimum bone manipulation [14].

The risk of developing MRONJ in in-treatment cancer patient undergoing dental implants is not only in the long term but it particularly increases in the short term. Therefore, dental implants are* contraindicated*, given the high degree of bone manipulation which is necessary for positioning the implant fixtures. Moreover, it can be added that the systemic health condition of a cancer patient could facilitate the rapid onset of a peri-implantitis, an additional great risk factor of MRONJ. Up to date, there are no published studies regarding the execution of pre-implant surgical treatment (e.g., guided bone regeneration) on in-treatment cancer patients. Notwithstanding a note of caution, it is the opinion of the authors that procedures relating to pre-implant treatment should be avoided in these patients, as well as the dental implant placement [1, 5, 14, 36].

All non-invasive dental treatments (e.g., restorative ) are not only considered as* indicated* but also of the utmost importance in reducing the spreading of infective processes [14, 37]. Notwithstanding this, some simple precautions prior to and during the dental examination should be taken: provide an antiseptic mouthwash to reduce the bacterial load in the oral cavity; do not use vasoconstricting anaesthetic; always work in isolation using a rubber dam, paying attention to the correct position of the clamps of the dam to avoid trauma to the oral mucosa. Moreover, during endodontic treatments, it is essential to avoid exceeding the limits of the root canal with endodontic instruments and root canal filling material [37].

As a non-invasive dental treatment, orthodontics is classified as an elective treatment and it is thus considered a* possible*, in absence of MRONJ cases published related to it. However, it has been suggested that orthodontic movements, which cause an increase in alveolar bone remodeling, in the cancer patients in-treatment may encourage the accumulation of drugs in the jawbone [38–40]. However, it must be underlined that cancer patient being treated with ONJ-related drugs will rarely request orthodontic treatment [14, 16, 39, 40].

Non-surgical periodontal therapy is strongly* indicated *and it should be carefully planned in order to remove regularly plaque and calculus and also periodically revise the oral health status of patient in-treatments [14–19, 25–27, 30–52]. Thus, it is essential to programme a four-month follow-up period for cancer patients in-treatment, without underestimating the contribution of the patient to the maintaining of effective oral hygiene at home and the self-screening of MRONJ[1, 4, 6, 14].

Dental prostheses in cancer patients in-treatment are* possible;* notwithstanding that nowadays there are few recommendations relating to this matter. Regarding the removable dentures, it is fundamental to reduce the pressure of the prosthesis on the oral mucosa and to maximize the stability, in order to avoid possible chronical trauma of oral mucosa [25, 53–56]. A four-month check-up period is desirable in cancer patients with removable dental prostheses, the aim of which is to constantly assess the fitting of the dentures and the absence of any area of compression and/or pressure ulcer, performing possible relining in soft resin, if needed [53–56]. Moreover, it is advisable that patients should not wear their dentures for approximately 8–12 hours per day (at least during the night).

Regarding the fixed prosthesis, it is important to pay particular attention to the biological width, avoiding the invasion of the junctional epithelium. Compatibly with the aesthetic needs of the patient, it would be ideal to provide a supragingival prosthetic margin, in order to facilitate check-ups and oral hygiene at home [57]. Dental treatments in cancer patients in the treatment phase are described in Table 3.

## 6. Non-cancer Patients in Treatment Phase

The dental management of a non-cancer patient already exposed to ONJ-related drugs is rather complex since it correlates with assessing risk according to variable gradients. These range from an undefined risk of MRONJ to a high risk of developing MRONJ. Indeed, the specific risk of MRONJ in the non-cancer patient varies according to the risk factors present; coexistence of more drug-related, systemic, and/or local risk factors is linked to various risk levels of MRONJ [14, 58].

Non-cancer patients are supposed to be divided into two categories regarding their risk to develop MRONJ; thereafter, from 6 months to within 3 years from the commencing of treatment, the patient who does not report other risk factors (systemic and/or local) will be classified in Category A and considered as a pre-treatment non-cancer patient at low risk of MRONJ.

Different in nature and variable is the assessment performed if the non-cancer patient has been in treatment for a period of time greater than 3 years or shorter than 3 years and simultaneously affected by systemic or local risk factors (Category B) (see Table 5); this patient will bear an incremental and indefinable risk of developing MRONJ, which is linked to one or more additional, reported systemic or local risk factors (see Table 6) [14, 17].

Surgical treatments (e.g., dental extractions, periodontal or endodontic surgery) aimed at removing infective outbreaks and the recovery of good oral health for Categories A and B are* indicated* procedures [14]. These procedures can be performed for non-cancer patients in-treatment in Category A, without applying specific medical and surgical protocols [59]. However, it will be necessary to use precautions with non-cancer patients in-treatment with Category B; these are similar to those described for the cancer patient in-treatment. For this reason, in patients in Category B, it is desirable to perform invasive treatments in combination with a prophylactic antibiotic therapy and to proceed tooth by tooth, particularly when the ONJ-related drug has not been suspended. Moreover, if available, it seems effective in applying low-level laser therapy (e.g., laser Nd:YAG) and APCs at the extraction site [32, 33]. After removing the sutures, it is of the utmost importance to perform periodic clinical-radiographic check-up (after 1, 3, 6, and 12 months) [25].

Elective invasive dental procedures, such as implantology and pre-implant bone surgery, in non-cancer patients in-treatment are not considered explicitly* contraindicated* but* possible *procedures, both for Categories A and B [60, 61]. Indeed, the risk/benefit ratio must be conscientiously assessed with the patient, who will be informed of the not definable risk of MRONJ: in the long term (e.g., risk of peri-implantitis) for Category A patients and in the long and short term (e.g., MRONJ related to the surgical procedures) for Category B. However, alternative treatment would be advised for patients included in Category B.

Promising results regarding the use of APCs during a surgical implant procedure have recently been reported for preventing MRONJ in non-cancer osteometabolic patients in-treatment by Mozzati et al., who have reported the absence of the development of MRONJ in a retrospective study one year after placing 1,267 implant placements on 235 patients, combined with the use of APCs [62].

As for cancer patients in-treatment, invasive and non-invasive dental treatments needed for the treatment of the prevention or the removal of inflammatory or infective lesions are mandatory in non-cancer patients in-treatments; in addition, prosthetic rehabilitation should contemplate the same recommendations [14, 25, 53–57].

Dental treatments of non-cancer patients in the in-treatment phase are described in Table 3.

## 7. Drug Suspension/Holiday

Regarding the latter, there has been much discussion in the literature about the validity of a temporary suspension of ONJ-related drugs; the aim of this biological window is to reduce the risk of an adverse event prior to surgical dental procedure. The temporary suspension of the ONJ-related drugs, the so-called drug holiday, must be compatible with basic pathologies and authorized by the prescriber. Such a suspension, when permitted, would be terminated preferably once the soft tissue had healed. Up to date, there is no scientific evidence which confirms the validity of the drug holiday, whether the drug/s are administered intravenously or orally, prior to the dental-alveolar surgery [1, 2, 5, 6, 14]. Specifically, the effects of BPs on the bone can be much prolonged over time, even after a single administration. The half-life of BPs is rather long, and they function by inhibiting osteoclast function for an unknown period of time. It can be hypothesized that suspending treatment could be associated with a reduction in the antiangiogenic effect of BPs on the periosteum and soft tissue [17, 63, 64]. This could contribute to vascularization improvement and encourage more rapid healing after surgery. Moreover, it could be useful in reducing the concentration of intravenous BPs at the extraction site in cancer patients, where their accumulation would increase tropism where there is extensive bone remodeling. Furthermore, about cancer patients in-treatment, any drug holiday should be considered to be a risky practice due to the possible progression of the oncological pathology and the absence of checking for bone-related events. In cancer patients in-treatment with different drugs by BP, suspending those is a desirable event. This will probably start from 7 days prior to any planned intervention (except for the Bevacizumab that should be suspended 6-7 weeks before), at least until the mucosal healing of the post-extraction site (see Table 4).The differing time periods are due to the fact that the well-known half-life of various MRONJ-related drugs is different. It is the experience of the authors that for the non-cancer patient included in category B, a drug holiday can already be considered useful one week before invasive dental procedures. However, this suspension is possible in cases where significant bone disequilibrium has not resulted, as assessed by the physician. BPs administration can resume 30–45 days after suspension, when the mucosa at the surgical site has healed (see Table 6). An interruption in BPs administration even months prior to surgery is suggested, where the systemic conditions of the patient permit this, and treatment is to be resumed after the total closing of any surgical wound. This approach, however, is based purely on expert opinion and it has not been yet validated in the literature. Since the beneficial effects of BPs in controlling basic diseases and related complications are well-known and while doubt remains regarding a BPs suspension, the patient must always be informed about the low predictability of such a suspension effect and the possible risks connected to the exacerbation of metabolic bone compensation. No drug suspension is necessary for the non-cancer osteometabolic patient in-treatment with Denosumab, given the latency period between subsequent Denosumab administrations, namely, every 6 months. It is appropriate to perform invasive procedures after 4 weeks from the last Denosumab administration and no later than the 6 weeks before the next administration, so as to ensure an adequate healing period. Should it be necessary to perform invasive procedures in a different time frame, it is advisable that these are planned within and not more than 45 days from subsequent administrations of Denosumab [6, 14, 52].

## 8. Conclusion

MRONJ is a rare but serious and highly debilitating disease since it can significantly compromise the patient's quality of life and reduce the compliance of the patients to AR/AA treatments. The number of cancer and non-cancer patients being treated with ONJ-related drugs and, therefore, the number of potentially adverse events seem constantly on the increase, also on the light of new drug related to ONJ. A multidisciplinary standardized approach with a sustained dialogue among clinicians involved in the treatment of patients at risk of MRONJ should be adopted in order to improve the efficacy of therapeutic strategies and to increase the patient's quality of life. The important role of the dentist in preventing the MRONJ, checking the local risk factors of MRONJ in pre-treatment and in-treatment patients, is evident.

Moreover, it is necessary to intervene in possible early signs of MRONJ for the secondary prevention. The application of such protocols of primary and secondary prevention, together with the dentists actions, the clinicians' sinergy, and the adequate awareness of the patient, is the key to implementing policies aimed at a common goal, that is, the reduction in outbreaks of MRONJ.

## Figures and Tables

**Table 1:** Oral risk factors of MRONJ [1].

*Oral risk factors*	
(i) Dental/periodontal infection	
(ii) Peri-implantitis	
(iii) Unfitting removable denture	
*Oral surgeries*	
(i) Dental extraction	
(ii) Dental implant surgery	
(iii) Endodontic surgery	
(iv) Periodontal surgery	
(v) Regenerative bone procedures	
*Anatomical conditions*	
(i) Torus and exostosis	
(ii) Pronounced mylohyoid ridge	

**Table 2:** Main dental treatments with respect to patients' categories in the pre-treatment phase with drugs related to ONJ.

Dental procedures on patients in the pre-treatment phase	Cancer patients	Non-cancer patients
Non-Surgical Procedures		
Restorative dentistry	Indicated	Indicated
Endodontic treatment	Indicated	Indicated
Orthodontic treatment	Possible	Possible
Periodontal treatments: oral hygiene and non-surgical treatments	Indicated	Indicated
Prosthesis	Possible	Possible
Surgical Procedures		
Dentoalveolar surgery	Indicated^*∗*^	Indicated
Preimplant bone surgery	Contraindicated	Possible^★^
Dental implant surgery	Contraindicated	Possible^★^
Periodontal/endodontic surgery	Indicated^§^	Indicated^§^

^*∗*^Advisable to wait for wound healing (4–6 weeks) before initiating antiresorptive or antiangiogenic treatment for cancer therapy. When treatment with ONJ-related drugs cannot be deferred, dentoalveolar surgery is indicated; in this case, the surgical protocol and medical treatment of oncological patients already in-treatment with MRONJ-related drugs will also be performed. ^§^To Perform only if any infective processes cannot be treated via periodontal/endodontic, non-invasive treatment. ^★^Advise the patient that the risk of MRONJ is indefinable in the long term.

**Table 3:** Main dental treatments in patients in-treatment phase with drugs related to ONJ.

Dental procedures on patients in-treatment phase	Cancer patients	Non-cancer patients	
		Category A	Category B
Non-Surgical Procedures			
Restorative dentistry	Indicated	Indicated	Indicated
Endodontic treatment	Indicated	Indicated	Indicated
Orthodontic treatment	Possible	Possible	Possible
Periodontal treatments: oral hygiene and non-surgical treatments	Indicated (every 4 months)	Indicated	Indicated
Prosthesis	Possible	Possible	Possible
Surgical Procedures			
Dentoalveolar surgery	Indicated	Indicated	Indicated
Preimplant bone surgery	Contraindicated	Possible^☆^	Possible^*∗*☆★^
Dental implant surgery	Contraindicated	Possible^☆^	Possible^*∗*☆★^
Periodontal/endodontic surgery	Indicated^§^	Indicated	Indicated^§^

°Follow the surgical protocol + adapt the flaps, avoid of tension and suture in order to prioritize healing of the wound. ^§^Perform only if any infective processes cannot be treated with non-invasive periodontal/endodontic procedures. ^☆^Advise the patient of an indefinable risk of MRONJ in the long term. ^★^Advise the patient of an indefinable risk of MRONJ in the short term.

**Table 4:** Drug suspension for cancer patients; it must be agreed upon with the oncologist and performed according to the table.

Drug holiday in cancer patients		
Active pharmaceutical ingredient	Last administration	Resume treatment
Bisphosphonate (AR)	At least 1 week before	4–6 weeks after
Denosumab (AR)	At least 1 week before	4–6 weeks after
Bevacizumab (AA)	At least 6-7 weeks before	4–6 weeks after
Sunitinib (AA)	At least 1 week before	4–6 weeks after
Everolimus (AA)	At least 1 week before	4–6 weeks after

**Table 5:** A classification of non-cancer patients already in treatment with MRONJ-related drugs.

Risk assessment of MRONJ in non-cancer patients	
Category A	Category B
(i) Patients eligible and not yet treated with ONJ-related medication (ii) Patients exposed to ONJ-related medication for less than 3 years, in absence of other systemic risk factors	(i) Patients exposed to ONJ-related medication for more than 3 years (ii) Patients exposed to ONJ-related medication for less than 3 years and in presence of other systemic risk factors (iii) Patients assuming BPs by IM^*∗*^

^*∗*^To date, there exists no data to distinguish groups of patients in treatment with zoledronate intravenous (annual medication intake) at greater or lesser risk of developing MRONJ.

**Table 6:** Drug suspension for non-cancer patients; it must be agreed upon with the prescriber and performed according to the table.

Drug holiday in non-cancer patients		
Active pharmaceutical ingredient	Last administration	Therapy resumption
Bisphosphonate^*∗*^ (AR)	1 week before	4–6 weeks after
Denosumab (AR)	No suspension^*∗∗*^	

^*∗*^Administered by more than three years or for less than three years and in the presence of other systemic risk factors; ^*∗∗*^suspension is not needed thanks to the latency between drug administrations. It is useful to perform invasive procedures between the first and the third month from the last administration, so as to ensure an adequate period for healing before the next dose.
